# Identification of New Potential Prognostic and Predictive Markers in High-Grade Osteosarcoma Using Whole Exome Sequencing

**DOI:** 10.3390/ijms241210086

**Published:** 2023-06-13

**Authors:** Raffaele Gaeta, Mariangela Morelli, Francesca Lessi, Chiara Maria Mazzanti, Michele Menicagli, Rodolfo Capanna, Lorenzo Andreani, Luca Coccoli, Paolo Aretini, Alessandro Franchi

**Affiliations:** 1 Section of Pathology, Department of Translational Research and of New Technologies in Medicine and Surgery, University of Pisa, 56126 Pisa, Italy; raffaele.gaeta@med.unipi.it (R.G.); rodolfo.capanna@unipi.it (R.C.); 2 Fondazione Pisana per la Scienza, San Giuliano Terme, 56017 Pisa, Italy; m.morelli@fpscience.it (M.M.); f.lessi@fpscience.it (F.L.); c.mazzanti@fpscience.it (C.M.M.); m.menicagli@fpscience.it (M.M.); p.aretini@fpscience.it (P.A.); 3 Department of Orthopedics and Trauma Surgery, Azienda Ospedaliera Universitaria Pisana, 56124 Pisa, Italy; l.andreani@ao-pisa.toscana.it; 4 Pediatric Hematology Oncology Unit, Azienda Ospedaliera Universitaria Pisana, 56126 Pisa, Italy; l.coccoli@ao-pisa.toscana.it

**Keywords:** osteosarcoma, genetics, whole exome sequencing, treatment, prognosis

## Abstract

Conventional high-grade osteosarcoma (OS) is the most common primary cancer of bone and it typically affects the extremities of adolescents. OS has a complex karyotype, and molecular mechanisms related to carcinogenesis, progression and resistance to therapy are still largely unknown. For this reason, the current standard of care is associated with considerable adverse effects. In this study, our aim was to identify gene alterations in OS patients using whole exome sequencing (WES) to find new potential prognostic biomarkers and therapeutic targets. We performed WES on formalin-fixed paraffin-embedded (FFPE) biopsy materials collected from 19 patients affected by conventional high-grade OS. The clinical and genetic data were analyzed according to response to therapy, presence of metastasis and disease status. By comparing good and poor responders to neoadjuvant therapy, we detected a clear prevalence of mutations in the *ARID1A, CREBBP, BRCA2* and *RAD50* genes in poor responders that negatively influence the progression-free survival time. Moreover, higher tumor mutational burden values correlated with worse prognosis. The identification of mutations in *ARID1A, CREBBP, BRCA2* and *RAD50* may support the use of a more specific therapy for tumors harboring these alterations. In particular, BRCA2 and RAD50 are involved in homologous recombination repair, and could thus be used as specific therapy targets of inhibitors of the enzyme Poly ADP Ribose Polymerase (PARP). Finally, tumor mutational burden is found to be a potential prognostic marker for OS.

## 1. Introduction

Conventional high-grade osteosarcoma (OS) is the most common primary malignant tumor of bone and affects about 2–3 cases/million/year, corresponding to 0.2% of all malignancies [1]. Current treatment of high-grade OS is based on a multidisciplinary approach that includes neoadjuvant chemotherapy, surgical excision of the primary tumor, evaluation of response to therapy in the surgical specimen and postoperative chemotherapy. Unfortunately, the outcome for patients presenting with metastatic disease and for those who have a poor response to initial treatment continues to be dismal, and the survival rate of patients is mainly linked to resistance to chemotherapy. Indeed, the prognosis of OS is related to the histologic response to neoadjuvant chemotherapy in terms of tumor necrosis and residual viable tumor, which can be evaluated in the surgical specimen [2,3]. However, recent studies suggest that in the current era of chemotherapy, prognosis may not be so closely related to post-treatment necrosis rate. Tsuda and colleagues found that optimal cut-off values of chemotherapy-induced necrosis may be lower than the current 90% for event-free survival (EFS) and overall survival (OS) [4]. In detail, other studies have indicated that a significant correlation between better OS and EFS is identified with a necrosis rate of 70% or more [5], suggesting the need for re-evaluation of the optimal cut-off value. Moreover, other parameters in addition to necrosis could be critical in assessing prognosis. Prabowo et al. proposed a scoring system predicting response to neoadjuvant chemotherapy based on integrating the histology with clinical data, e.g., gender, age and increased tumor size after neoadjuvant chemotherapy [6]. Other authors found that metastasis at diagnosis, tumor size larger than 10 cm and metastasis development are factors that are significantly associated with poor survival, and function as independent prognostic factors [7].

OS has a complex karyotype, and the molecular mechanisms related to carcinogenesis, progression and resistance to therapy are still largely unknown. The cause of the high genomic instability has been hypothesized to be chromothripsis, i.e., ‘the shattering of one or a few chromosomes into small fragments that are stitched together in a random order and orientation’, which occurs in about 30% of OSs [8]. Chromothripsis may be caused by physical chromosomal damage (e.g., ionizing radiation) or by the progressive shortening after many cell divisions of telomeres, which are responsible for maintaining the genomic integrity in normal cells. As a result of these events, driver genes (i.e., cancer-causing genes) may appear due to several mechanisms, including increased copy number (amplification of oncogenes), decreased copy number (deletion of tumor suppressor genes), the juxtaposition of coding sequences from two genes (fusion oncoprotein) or the bringing together of an intact gene with the promoter of a different gene, resulting in dysregulation of its expression [9]. Another phenomenon present in OS is kataegis, which is a result of elevated mutation prevalence over regions in chromosomes. In OS, the prevalence of kataegis, estimated on the basis of a small cohort of samples, was high, ranging between 50 and 85% [10].

Few recurrent alterations have been identified in OS. Certain hereditary syndromes predispose to OS, such as Li–Fraumeni syndrome (mutations in *TP53* or, less frequently, *CHEK2*) [11] and retinoblastoma (mutations in *RB1*) [12]. Moreover, syndromes associated with mutations in RecQ-like helicases (e.g., Rothmund–Thomson syndrome, RAPADILINO syndrome, Baller–Gerold syndrome, Werner syndrome and Bloom syndrome) have an increased risk for OS [13]. In OS not associated with hereditary syndromes, mutations in the p53 or Rb pathways are the most common genetic alterations [14]. Another determining factor for chromosome stability is the *BRCA* pathway, which is responsible for DNA repair. It has been shown that mutations, genomic instability and loss of heterozygosity resulting in *BRCA1/2* inactivation occur in 91% and 78% of OSs, respectively [15].

In this study, we performed whole exome sequencing (WES) analyses to identify clinically relevant genetic alterations in OS that are involved in tumor progression, development of metastasis and resistance to cytotoxic therapy, and to identify new therapeutic approaches to offer patients a tailored treatment based on specific genetic abnormalities.

## 2. Results

### 2.1. Patient Characteristics

Twenty-two patients were enrolled in this study. In three cases, the biopsy samples yielded low-quality or insufficient DNA, making it difficult to perform the WES. Thus, the final number of patients included in the study is 19. Their clinico–pathological characteristics are shown in Table 1.

Patients were treated according to the AIEOP ISG/OS-2 protocol, which consists of the combined use of high-dose methotrexate (HDMtx), doxorubicin (adriamycin) and cisplatin for neoadjuvant chemotherapy (“MAP regimen”). Patients who had a poor histological response received four cycles of high doses of ifosfamide (“MAPI regimen”) and Mepact (mifamurtide). Overall, the evaluation of chemotherapy-induced tumor necrosis in the surgical specimens showed a slight prevalence of GR over PR (ten vs. nine). The follow-up period ranged between 6 and 54 months (mean: 31.6 months). Overall, six patients developed metastases (mean time: 25.9 months ± 15.5 SD), while one suffered from local recurrence after 17 months. Four patients (21%) died of disease.

### 2.2. Landscape of Genomic Alterations

We successfully performed WES on 19 pre-treatment tumor tissue samples. After several preprocessing and filtering steps, a total of 52,701 somatic single nucleotide variants (SNVs), including 38,123 missense, 1567 nonsense and 1912 somatic indels were identified. 

The analysis performed with Maftools allowed us to stratify the mutations in driver genes by type, protein impact and the single nucleotide variant class (SNV class). We also emphasized the “top mutated genes” and differences in the frequency of mutation in single genes between the groups. 

**Figure 1:**
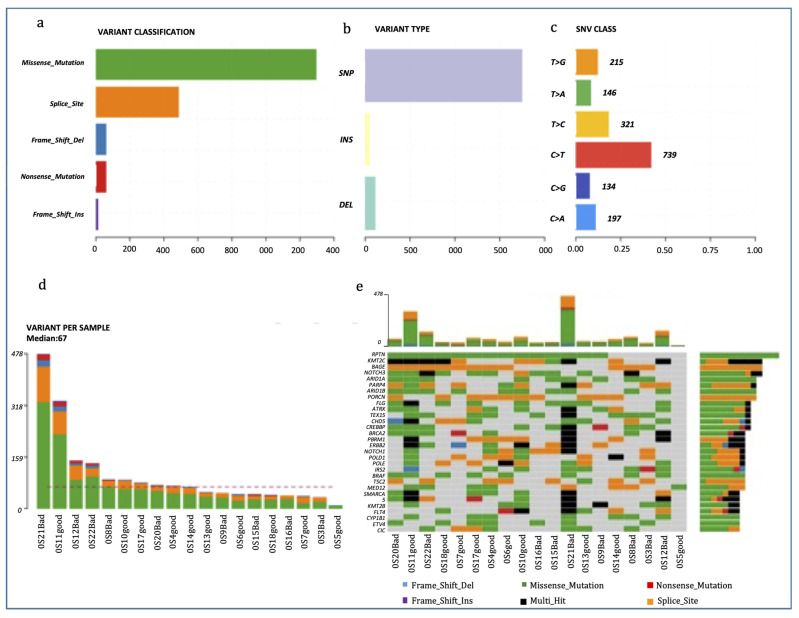
Mutational profiling of the OS cohort. (**a**–**c**): Counts of each variant classification (**a**), variant type (**b**) and each single nucleotide variant (SNV) classification (**c**). (**d**) Counts of variants per sample. (**e**) The 30 most mutated genes and the number of mutations stratified per gene and per sample.

Figure 1 is a summary of the alterations in the OS samples. The most frequent genetic variations were missense mutations, and the type of variation most frequently found was single nucleotide polymorphisms (SNPs), with C > T being the most frequent single nucleotide variation. OS21 presented the highest number of genetic alterations *(n* = 478) (Figure 1). The most frequently mutated gene was *RPTN* (mutated in 14 out of 19 patients), followed by *KMT2C*, *BAGE* and *NOTCH3* (11/19). We identified *BRCA2* mutations in 8 samples (42.1%), while 4 OSs presented *BRCA1* mutations. *BRCA2* alterations were mainly missense mutations followed by multi-hit and nonsense mutations, while all *BRCA1* alterations were missense mutations. Interestingly, tumors carrying *BRCA2* mutations had a significantly higher tumor mutation burden than wild type OSs (mean of 172.25 ± 54.45 SE vs. 55.27 ± 6.37, *p* = 0.016; Mann–Whitney U test).

### 2.3. Genomic Characteristics in OS with Different Treatment Responses

**Figure 2:**
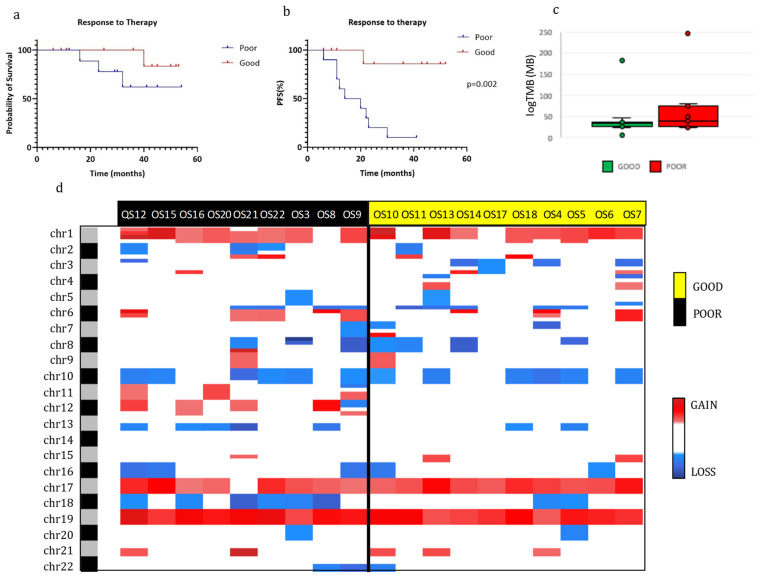
Molecular characterization of osteosarcomas in good and poor responders. (**a**,**b**): Kaplan–Meier overall survival (OS) (**a**) and progression-free survival (PFS) (**b**) curves of OS patients according to chemotherapy response. (**c**) Tumor mutational burden (TMB) of good and poor responders. (**d**) Heatmap of individual copy number region profiles between poor (*n* = 9) and good (*n* = 10) responder groups (red for chromosome gains and blue for losses). Abbreviations: Good, good responders; Poor, poor responders.

To characterize our OS population according to treatment response, we first correlated the overall survival and the PFS with treatment response (Figure 2a,b). Good responders (GR) had a higher probability of survival than poor responders (PR), even if this difference was not significant. However, a good response to therapy was a statistically significant positive prognostic factor for PFS compared with a poor response (*p* = 0.002).

Additionally, the DNA copy number alterations (CNAs) analysis in the GR and PR groups (Figure 2d) revealed a slightly higher presence of insertions/deletions in the PR when compared with the GR. In particular, a significantly higher percentage of alterations in chromosome 12 (gain) and 18 (loss) were present in PR than in GR (chr12: 5/9 vs. 0/10; chr18: 6/9 vs. 2/10, respectively). No other significant differences in CNAs between the two groups were found and there were also no significant differences in tumor mutational burden (Figure 2c).

**Figure 3:**
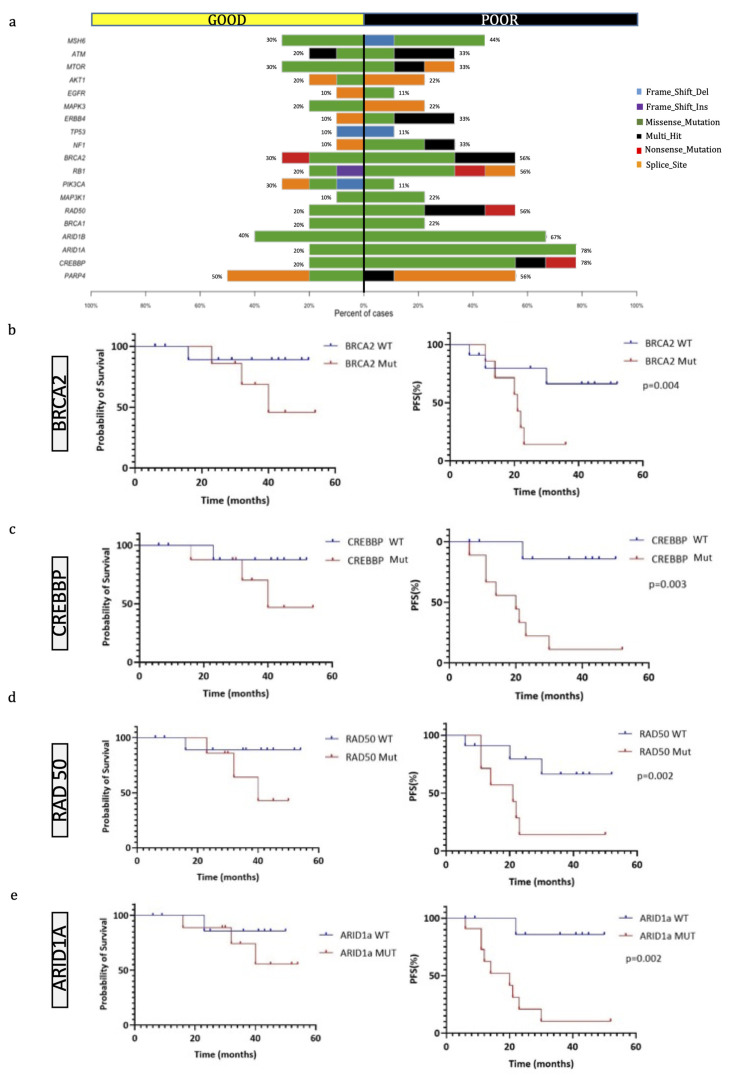
Genes most frequently mutated in OS patients. (**a**) Co-bar plot of the genes significantly discriminating good and poor responders. Bars represent the % of samples in which gene mutations have been identified and colors indicate the type of mutation. (**b**–**e**) Kaplan–Meier overall survival (OS) curves on the left and progression-free survival (PFS) curves (on the right) of OS patients according to the mutational status of *BRCA2* (**b**), *CREBBP* (**c**), *RAD50* (**d**), and *ARID1a* (**e**).

Some genes were more frequently mutated in the PR group than in the GR group, as highlighted by the co-bar plot representation in Figure 3a. In particular, in PR patients, there was a prevalence of mutations in *BRCA2*, *RB1*, *RAD50*, *ARID1A*, *ARID1B* and *CREBBP*. Differences in the most frequently mutated genes were also detected between GR and PR. In GR, there were alterations in *RPTN* (80%), *BAGE* (70%), *POLD1* (70%) and *PORCN* (70%), while in PR there were alterations in *CREBBP* (78%), *KMT2C* (67%), *ARID1A* (78%) and *BRCA2* (56%) (Figure S1).

Using pairwise Fisher exact tests, we also analyzed the somatic interactions between the most frequently mutated genes in the two groups, both with co-occurrence and mutual exclusivity. In the GR group, we found a significant co-occurrence of certain genes with *ATRX*, whose role in OS has not yet been clearly defined (Figure S2A). Conversely, a remarkable finding from the analysis of PRs was the frequent involvement of genes which have already been described as altered in OS, such as *BRCA2, RB1, NOTCH3* and *PARP4*. In some cases, there was a strong co-occurrence (such as between *PARP4* and *RAD50*), while *NOTCH3* and *KMT2B* mutations were mutually exclusive (Figure S2B).

### 2.4. Genomic Characteristics of OS according to Disease Status

**Figure 4:**
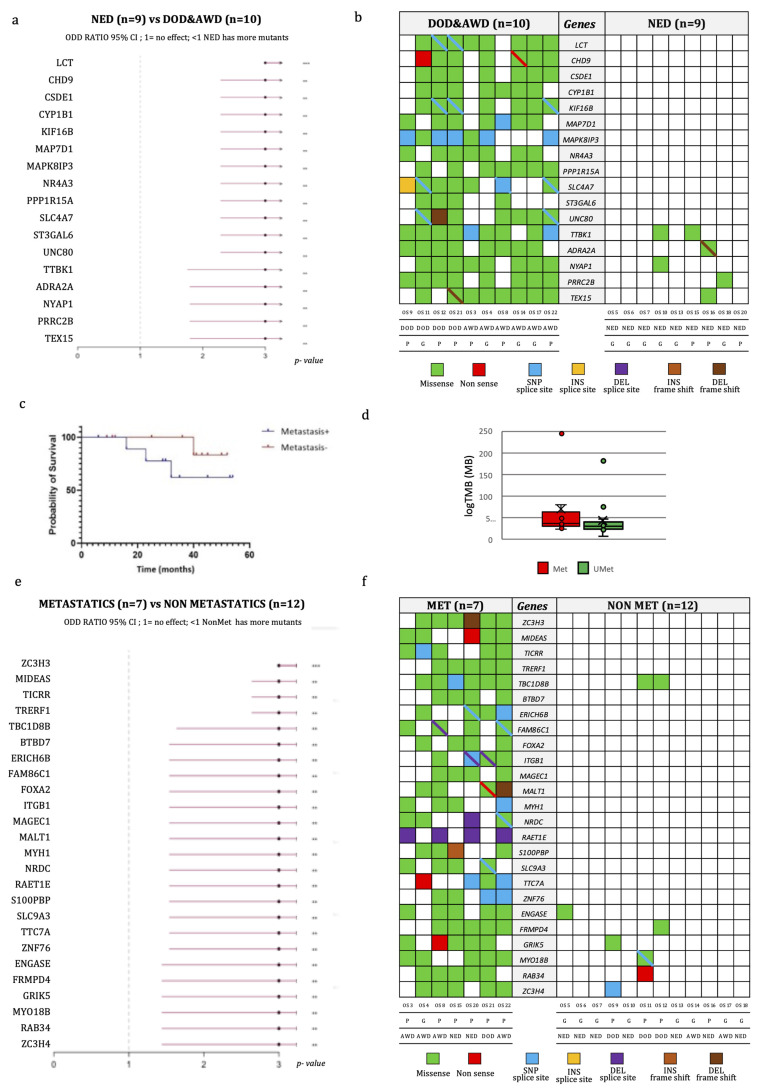
Genomic characteristics of OS according to disease progression. (**a**) Odds-ratio plot of differentially mutated genes discriminating patients with disease progression from those with no evidence of disease at last follow-up. (**b**) Variant types of differentially mutated genes, listed in the central gray column, discriminating patients with no evidence of disease at last follow-up (**on the right**) from the other patients (**on the left**). Each cell represents a patient, whose code, response to treatment and disease status is reported at the bottom. The different colors represent the variant types as indicated in the legend. The diagonal bar indicates the co-presence of 2 variants: one from the color of the cell, and the other from the color of the bar. (**c**) Kaplan–Meier overall survival (OS) curve of OS patients according to the presence of metastasis. (**d**) Tumor mutational burden (TMB) of metastatic and non-metastatic OS patients. (**e**) Odd-ratio plot of differentially mutated genes discriminating metastatic from non-metastatic patients. (**f**) Variant types of differentially mutated genes, listed in the central gray column, discriminating metastatic (**on the left**) from non-metastatic patients (**on the right**). Each cell represents a patient, whose code, response to treatment and disease status is reported in the bottom. The different colors represent the variant types. The diagonal bar indicates the co-presence of 2 variants, one indicated by the color of the cell and the other by the color of the bar. ** *p*-value ≤ 0.01: *** *p*-value ≤ 0.001. Abbreviations: P, poor responders; G, good responders; AWD, alive with disease; NED, non-evidence of disease; DOD, dead of disease; INS, insertion; DEL, deletion.

Concerning the genetic findings, factors that negatively influenced the PFS interval were mutations in the *BRCA2* (*p* = 0.04), *ARID1A* (*p* = 0.002), *CREBBP* (*p* = 0.003) and *RAD50* (*p* = 0.002) genes (Figure 3b–e), even if these mutations did not affect the overall survival time. We then compared the PFS time of patients with tumors harboring either *BRCA1* or *BRCA2* mutations with those possessing wild-type OSs, and we observed that the group with *BRCA1/2* mutated OSs had a significantly lower PFS time (*p* = 0.02, log-rank test). Overall, we identified 17 mutated genes discriminating patients with no disease progression from patients either alive with the disease or dead from the disease (Figure 4a,b), which have not been implicated in the biology of OS before. 

We then investigated the correlation of tumor mutational burden (TMB) with prognosis. The TMB median value was 33.6 (min–max: 6.3–183.0) and 39.2 (min–max: 23.7–247.7) per megabase in GRs and PRs, respectively, even if this difference was not statistically significant (Figure 2c). However, when we divided our cohort of OSs in high and low TMB by the median value and by the 75th percentile, some relevant differences emerged. In specific, patients with low TMB had a significantly better PFS (*p* = 0.01, log-rank test) and overall survival (*p* = 0.02, log-rank test) using the 75th percentile as cutoff, while the difference was borderline for PFS and absent for overall survival with the cutoff at the median value (*p* = 0.051 and *p* = 0.22, respectively; log-rank test).

Additionally, patients without metastases (*n* = 12) at the last follow-up exhibited a higher probability of survival than those who developed metastases (*n* = 7), even if this difference did not reach statistical significance (Figure 4c). The correlation of TMB with the presence of metastases was also investigated (Figure 4d). We found 25 mutated genes discriminating metastatic from non-metastatic patients (Figure 4e,f). None of these genes were previously found to be implicated in OS progression or prognosis. The difference in the TMB median value among metastatic (median: 37/MB; min–max: 22–243/MB) and non-metastatic (median: 29/MB; min–max: 6–180/MB) patients was not significant. 

## 3. Discussion

OS is a rare tumor that still maintains a severe prognosis. The impact of the disease is dramatic as it predominantly affects individuals of pubertal age, and its treatment has a deep effect on patient lives. Thus, it is critical to fully investigate the molecular mechanisms of OS to understand the basis of tumor progression and response to therapy. In the present study, interesting results emerged by comparing the genetic data of OSs with GR and PR to neoadjuvant treatment. Regarding CNVs, the PR group exhibited a higher frequency of chromosome 12 amplification and chromosome 18 loss. In accordance with previous studies, chromosome 18 is frequently lost in OS specimens, but no studies have identified a distinct tumor suppressor gene. Relevant oncogenes, such as MDM2 and CDK4, are instead located on chromosome 12, which are amplified with region gain [16].

Further, we noted that patients with poor response to chemotherapy, and thus with the worst prognosis, showed a higher rate of mutations of *CREBBP* (78% versus 20%), *ARID1A* and *1B* (67% versus 40%), *BRCA2* (56% versus 30%), *RB1* (56% versus 20%) and *RAD50* (56% versus 20%) compared to GRs. The CREB binding protein is a histone acetyltransferase and a transcriptional coactivator since it connects other proteins that start the transcription process. Its mutations are associated with several diseases, such as Rubinstein–Taybi syndrome 1 [17] and Menke–Hennekam syndrome 1 [18]. In bone cancer, mutations of *CREBBP* have been reported in NGS studies [19], but its role has not been fully investigated yet. Our findings suggest a possible association of *CREBBP* mutations with poorer response to therapy in OS patients.

Two other frequently mutated and clinically actionable genes are AT-rich interactive domain-containing protein 1A (*ARID1A*) and 1B (*ARID1B*), which encode proteins that are part of the large ATP-dependent chromatin remodeling complex SNF/SWI, a component required for the transcriptional activation of genes normally repressed by chromatin. *ARID1A*, in particular, has been shown to function as a tumor suppressor in various malignancies, such as ovarian clear cell, ovarian endometrioid and uterine endometrioid carcinomas [20]. WES performed on 21 patients with osteosarcoma identified *ARID1A* mutations in 43% of cases, but its biological role in OS is not clear [21]. Fatema et al. noted that human OS cell lines with *ARID1A* loss in vitro (CRISPR/Cas9) developed faster tumorigenesis [22]. Later, they observed that *Arid1a*-mutant mice develop tumors and die, on average, 13 weeks earlier than the wildtype cohort, indicating that *ARID1A* probably plays a significant role in OS genesis, progression and metastasis. Our data, along with previous studies, demonstrate that *ARID1A* and *1B* may have a role as tumor suppressors in OS progression, and may represent useful prognostic markers and potential therapeutic targets.

The high prevalence of *BRCA2* mutations in the PR patients compared to the GR patients is a significant finding from our study. Mutations in the *BRCA* pathway, which is responsible for DNA repair, have been previously reported in OS [15]; however, we have shown that they occur at a different rate in patients with different responses to therapy. This observation is crucial since *BRCA* mutations cause defects in the homologous recombination pathway that lead to defects in DNA repair mechanisms which can be treated by Poly-(ADP-Ribose)-Polymerase1,2 inhibitors (PARPi).

It appears that the use of PARPi might not be limited to tumors with mutation in the *BRCA1/2* tumor suppressors since Zoumpoulidou et al. [23] documented highly penetrant PARPi hypersensitivity following mutations of *RB1* (a negative regulator of the cell cycle). The authors concluded that the data provide evidence that *RB1* status is a predictor of single-agent PARPi sensitivity in osteosarcoma-derived cells, with sensitivity levels comparable to that of *BRCA2*-mutated cancer cells. In our population, we observed a high proportion of mutations in *RB1* in poor- versus good-responders (56% versus 20%). In addition, no nonsense mutations were present in the GR group, whereas they were present in the PR patients. If, therefore, mutations in *RB1* are indeed associated with clinical hypersensitivity to PARP inhibitors, the mutational study of this gene could represent a new target strategy for the treatment of OS and an important predictive factor. 

Another frequently mutated gene in patients with poor histologic response to chemotherapy is *RAD50*, whose mutations were identified in 56% of PRs compared to 20% of GRs. It is part of a complex composed of *MRE11-RAD50-NBS1* (MRN) that plays an important role in DNA double-strand break regeneration via homologous recombination and non-homologous end-joining pathways as well as in telomere maintenance, DNA replication, and cell cycle checkpoints [24]. Notably, MRN complex mutation-related homologous recombination deficiencies may be capable of sensitizing cancer cells to PARP inhibitor therapy, and might thus be applicable for providing a predictive biomarker of PARP inhibitor-based therapy in OS [25].

Moreover, interesting data emerged regarding the TMB, defined as a total number of non-synonymous somatic mutations in coding areas per tumor genomic megabase. The prognostic role of TMB in solid tumors is debated. In our study, high TMB was associated with poorer survival both in terms of PFI and overall survival when the 75th percentile was used as the cutoff value. In agreement with our results, high TMB was associated with worse disease-free survival and overall survival in a retrospective study of 90 patients who had surgery for early-stage non-small cell lung cancer and who did not receive neo-adjuvant treatment (chemotherapy or immunotherapy) [26]. Similarly, a pooled analysis of 103,078 patients with cancer (including non-small cell lung cancer, ovarian cancer, urothelial carcinoma, melanoma, head and neck cancer, glioma, colorectal cancer, breast cancer, hepatocellular carcinoma, myeloma and studies including diverse cancer types) concluded that high TMB is associated with worse survival in patients with non-small cell lung cancer, melanoma, myeloma, hepatocellular carcinoma and breast cancer, but it is associated with better survival in patients with ovarian cancer [27]. The authors here concluded that the prognostic role of TMB is possibly dependent on the tumor type. In contrast, Xie L. et al. recently reported a positive correlation between high TMB and a long PFS and overall survival in a series of 31 OS patients [28].

In addition, we found two sets of mutations discriminating between OS patients with and without disease progression and between patients developing metastasis and patients that are metastasis free. These genes have not been implicated in OS before, and further studies are needed to elucidate their possible role in tumor progression of OS and as predictors of clinical outcome. 

The main limitations of this study are the small number of patients analyzed due to the rarity of OS and a relatively short median follow-up. In addition, both tumor cohorts with poor and good responses to neoadjuvant treatment presented mutations in *ARID1A, CREBBP, BRCA2* and *RAD50*, although with significantly different frequencies. Therefore, these markers are not yet ready to guide therapeutic procedures at diagnosis, but they could rather be useful to adjust adjuvant rather than neoadjuvant treatment in case of a poor response. Further studies, possibly involving multiple centers, to increase the number of subjects and lengthen the follow-up time, are warranted to confirm our findings.

## 4. Materials and Methods

### 4.1. Case Selection

In this study, we enrolled patients <40 years of age affected by the primary high-grade conventional OS. In all cases, we analyzed the biopsy material before chemotherapy treatment. The material was fixed in 4% buffered formalin, processed and embedded in paraffin (formalin-fixed paraffin-embedded, FFPE). After neoadjuvant chemotherapy and surgical resection of the tumor, a histological evaluation of treatment-induced tumor necrosis was performed on the specimen [29]. Patients with tumors showing ≥90% of necrosis were classified as good responders (GRs), whereas patients with tumors showing <90% of necrosis were classified as poor responders (PRs).

### 4.2. DNA Extraction and Quantification

The genomic DNA extraction was performed using a Maxwell^®^ 16 Instrument (Promega), with the Maxwell^®^16 FFPE Tissue LEV DNA Purification Kit (Promega, Madison, WI, USA). The starting material for every FFPE block consisted of 3 sections of 10 µm each. 

### 4.3. Whole Exome Sequencing

The whole exome library preparation was performed using the Illumina DNA Prep with Enrichment Kit (Illumina, San Diego, CA, USA) following the manufacturer’s procedure. The libraries were run on a NextSeq 550 High Output Cartridge (300 cycles) with an average coverage of 100×. Finally, paired-end sequencing was performed on a NextSeq 500 system (Illumina, San Diego, CA) with 151 bp sequencing.

### 4.4. Bioinformatic NGS Analysis 

A typical workflow of WES analysis consists of the following steps: raw data QC, preprocessing, mapping, post-alignment processing, variant calling, annotation and prioritization. The raw data generated from NextSeq500™ were converted using the Bcl2toFastq tools provided by Illumina. The primary data analysis of exomes was performed by using the SeqMule pipeline [30], which implements all the methods described.

To obtain a picture of somatic mutations and to overcome the problem of missing respective healthy tissues, we used Pisces [31]. Pisces is unique primarily because it excels in the difficult and common situations where no matched normal sample exists for a given tumor sample. 

The resulting VCF files were annotated using the Illumina Variant Interpreter (https://variantinterpreter.informatics.illumina.com/home, accessed on 12 September 2022). Subsequently, to obtain the rare variants, somatic variants reported in the non-cancer database gnomAD v3 [32] with a minor allele frequency of ≥ 0.01 were excluded. 

To improve the selection of variants, we also used other filters: quality score ≥ 10, read depth ≥ 30 and protein impact involving missense, nonsense, frameshift and splice disrupt mutations (i.e., the variants that affect the protein structure or regulatory regions). The degree of pathogenicity of the missense variants was evaluated in silico by using Varsome (https://varsome.com, accessed on 15 September 2022). 

The frequency and type of mutations were investigated using the R package MAFtools [33]. Additionally, Integrative Genome Viewer was used to manually check for specific mutations in the files created after alignment with the reference genome.

Copy number alterations were estimated by CNVkit [34] and summarized using the CNApp [35] with default cutoffs.

### 4.5. Statistical Analysis

All statistical tests were performed using the SPSS software (release 20.0). The endpoints considered in this study were the evaluation of the progression-free survival (PFS) and overall survival defined as the time from treatment initiation to the time of progression/last follow-up and death/last follow-up, respectively. The analysis of survival was modeled using the Kaplan–Meier method and analyzed by the log-rank test. Differences between groups of categorical data were analyzed using either Fisher’s exact test or Pearson’s χ^2^. The Mann–Whitney U test was used to compare two groups of unpaired values. To identify statistically significant localizations of CNAs on chromosome arms, we used the Student’s *t*-test with multiple testing corrections by BH method [36]. All *p* values resulted from the use of 2-sided statistical tests. *p* values < 0.05 were considered to indicate statistically significant differences.

## 5. Conclusions

WES investigations allowed an in-depth analysis of the genetic characteristics of a series of OSs, a rare tumor type that still has a poor prognosis in a significant proportion of cases. The most promising results concern the identification of mutations that are associated with worse prognosis and better response to therapy, such as *BRCA*, *CREBBP*, *ARID1A* and *RAD50*. These biomarkers could be critical for early prognosis assessment and to provide a more specific treatment for subjects with tumors harboring these mutations. Since several studies have shown that neoplasms harboring *RAD50*, *BRCA* and *RB1* mutations are susceptible to a group of pharmacological inhibitors of the enzyme poly ADP ribose polymerase (PARP), patients whose tumors present these mutations might benefit from personalized targeted therapy. On the other hand, patients with tumors showing a mutational profile associated with a better prognosis may avoid heavy non-specific therapies leading to several side effects. Further studies are certainly necessary to clarify the association between TMB and survival; this may lead to the use of TMB as a prognostic marker in predicting the outcome of patients with OS.

## Figures and Tables

**Table 1:** Patient characteristics in this study (Abbreviations: AWD = alive with disease; NED = no evidence of disease; DOD = dead of disease; MAP = Methotrexate, Doxorubicin, and Cisplatin; MAPI = MAP plus ifosfamide).

Patient nr.	Age at Diagnosis	Sex	Histotype	Site	Drug Treatment	Surgical Treatment	Necrosis	Metastases at Diagnosis	Follow Up
							(Huvos Grade)		(Months after First Diagnosis)
OS3	10	M	High-grade osteoblastic	Distal femur (left)	MAPI + Mepact	Wide excision	80%	No	Pulmonary metastases;
							(Grade II)		AWD (29)
OS4	10	F	High-grade, NOS	Distal femur (right)	MAP + Mepact	Wide excision	95%	Pulmonary and bilateral bone lesions	Pulmonary metastases;
							(Grade III)		AWD, in therapy (9)
OS5	12	M	High-grade osteoblastic	Distal femur (left)	MAP	Wide excision	95%	No	NED (45)
							(Grade III)		
OS6	13	M	High-grade, NOS	Distal femur (left)	MAP	Wide excision	98%	No	NED (43)
							(Grade III)		
OS7	13	F	High-grade fibroblastic/chondroblastic	Distal femur (right)	MAP + Mepact	Wide excision	95%	No	NED (36)
							(Grade III)		
OS8	14	M	High-grade osteoblastic	Distal femur (right)	MAP	Wide excision	70%	No	Several local recurrences;
							(Grade II)		AWD, in therapy (54)
OS9	14	M	High-grade osteoblastic	Proximal humerus (right)	MAP	Amputation	45%	No	DOD (16)
							(Grade I)		
OS10	14	M	High-grade osteoblastic	Proximal humerus (right)	MAP	Wide excision	99%	No	NED (52)
							(Grade III)		
OS11	15	F	High-grade fibroblastic	Distal femur (left)	MAP	Wide excision	95%	No	DOD (40)
							(Grade III)		
OS12	15	M	High-grade osteoblastic	Proximal tibia (right)	MAP	Wide excision	80%	No	DOD (23)
							(Grade II)		
OS13	16	F	High-grade osteoblastic	Distal femur (left)	MAPI + Mepact	Wide excision; subsequent amputation	98%	Lung (bilateral) and controlateral thigh lesion	NED (50)
							(Grade III)		
OS14	16	M	High-grade fibroblastic	Proximal fibula (left)	MAP + Mepact	Wide excision	98%	No	In therapy (9)
							(Grade III)		
OS15	18	M	High-grade osteoblastic	Distal femur (right)	MAP	Wide excision	80%	No	Pulmonary metastases;
							(Grade II)		NED after chemotherapy (35)
OS16	19	M	High-grade, NOS	Distal femur (right)	MAPI + Mepact	Wide excision	75%	No	NED (41)
							(Grade II)		
OS17	19	M	High-grade, NOS	Distal femur (right)	MAP + Mepact	Wide excision	95%	No	In therapy (6)
							(Grade III)		
OS18	20	M	High-grade, NOS	Proximal tibia (left)	MAP	Wide excision	98%	No	NED (25)
							(Grade III)		
OS20	22	M	High-grade osteoblastic	Distal femur (right)	MAPI + Mepact	Wide excision	80%	No	Pulmonary metastases;
							(Grade II)		NED after chemotherapy (30)
OS21	27	M	High-grade chondroblastic	Distal femur (right)	MAP	Wide excision	20%	No	DOD after pulmonary tumor thromboses (32)
							(Grade I)		
OS22	34	F	High-grade osteoblastic	Distal femur (right)	MAP	Wide excision	60%	No	Pulmonary metastases;
							(Grade II)		AWD (45)

## Data Availability

The data that support the findings of this study are available from the corresponding author upon reasonable request.

## Supplementary Materials

The following supporting information can be downloaded at https://www.mdpi.com/article/10.3390/ijms241210086/s1.
