# Supplementary material for: Identification of New Potential Prognostic and Predictive Markers in High-Grade Osteosarcoma Using Whole Exome Sequencing
Source: Int J Mol Sci. 2023 Jun 13;24(12):10086. doi: 10.3390/ijms241210086 (PMC10298506; doi:10.3390/ijms241210086)
Supplement: Supplementary file 1 [file ijms-24-10086-s001.zip › ijms-2436824-supplementary.pdf]

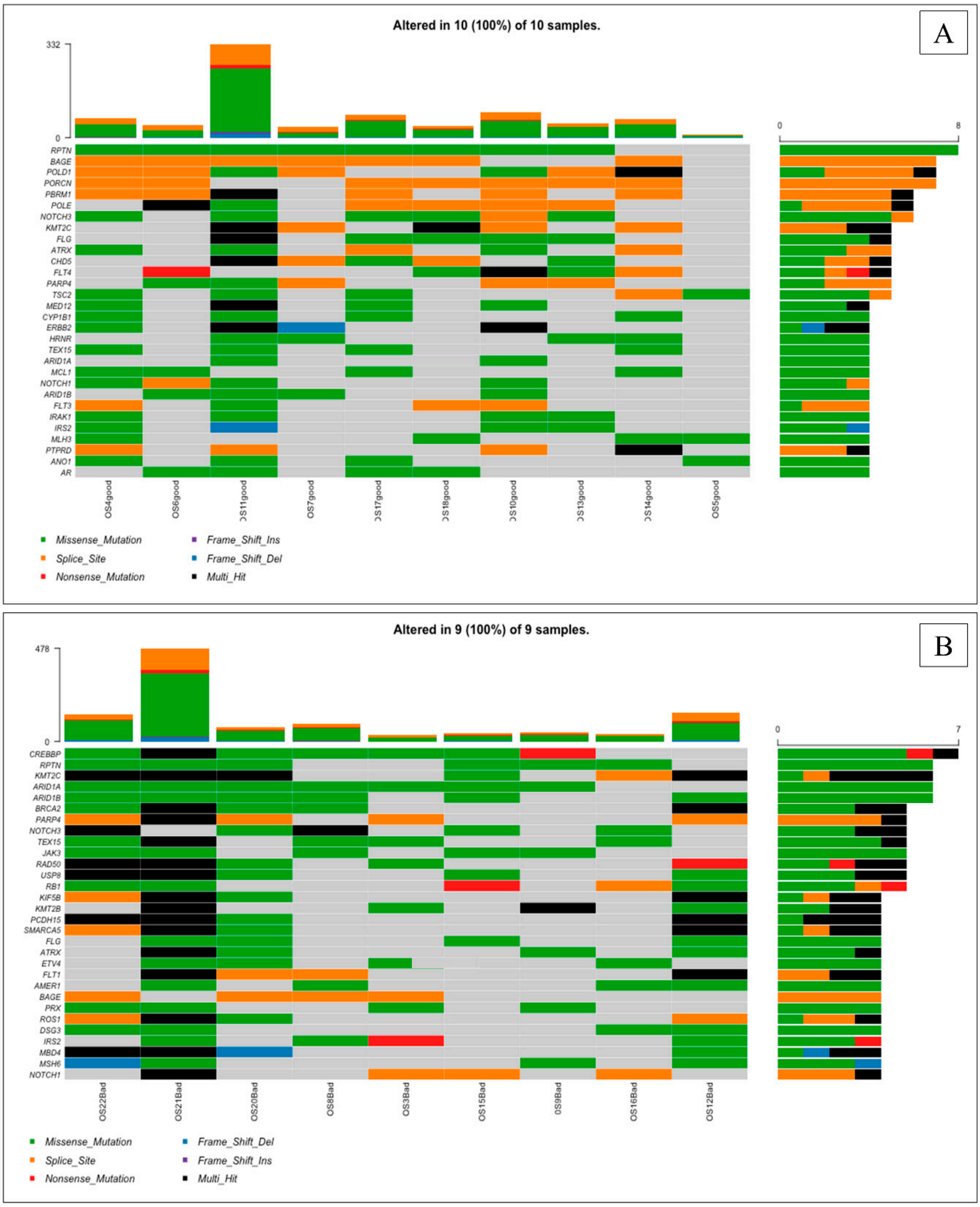

Figure S1. The 30 most mutated genes and the number of mutations stratified per gene and per sample in the GR (**A**) and PR (**B**).

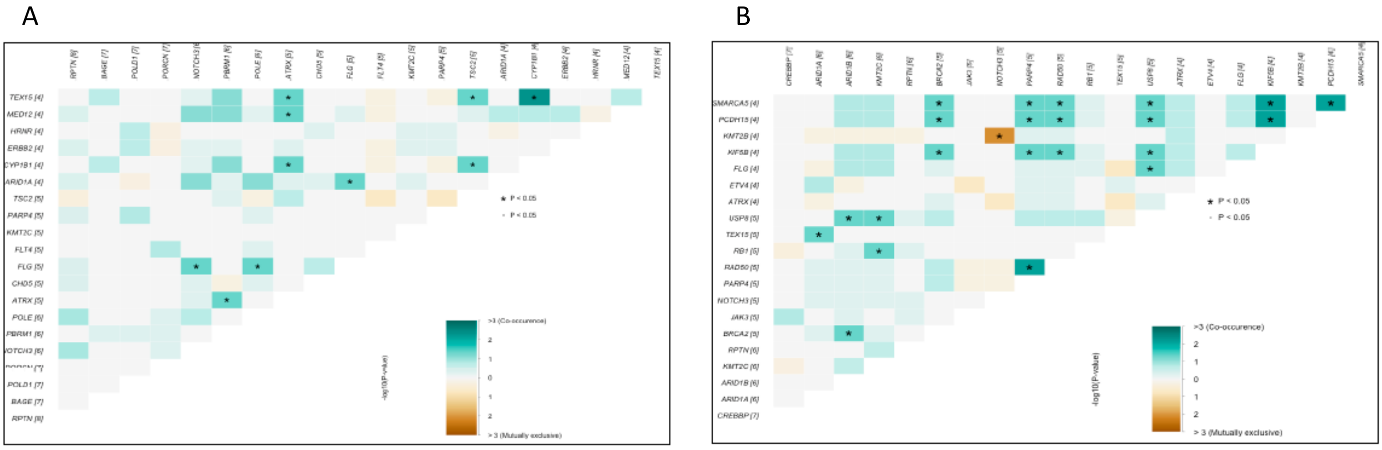

Figure S2. Somatic interactions between the most frequently mutated genes in GR (**A**) and PR (**B**) groups, both co-occurrence and mutually exclusive, using pairwise Fisher exact tests.
